# Acupuncture Treatment for Bortezomib-Induced Peripheral Neuropathy: A Case Report

**DOI:** 10.1155/2011/920807

**Published:** 2011-03-09

**Authors:** Ting Bao, Ruixin Zhang, Ashraf Badros, Lixing Lao

**Affiliations:** ^1^Marlene and Stewart Greenebaum Cancer Center, The University of Maryland School of Medicine, University of Maryland, Baltimore, MD 21201, USA; ^2^Center for Integrative Medicine, The University of Maryland School of Medicine, University of Maryland, Baltimore, MD 21201, USA

## Abstract

Peripheral neuropathy is a common and severe dose-limiting side effect of the chemotherapy agent, bortezomib, in multiple myeloma patients. Treatment with narcotics, antidepressants, and anticonvulsants has limited response and potential significant side effects. Acupuncture has been reported to be effective in treating diabetic neuropathy and chemo-induced peripheral neuropathy. There has not been report on the effect of acupuncture in treating bortezomib-induced peripheral neuropathy specifically. Here, we report a successful case of using acupuncture to relieve bortezomib-induced peripheral neuropathy symptoms.

## 1. Introduction

Peripheral neuropathy in patients with multiple myeloma occurs as both disease- and treatment-related complication in the relapsed and newly diagnosed setting. Bortezomib is a novel agent in treating multiple myeloma [1]. Bortezomib-induced peripheral neuropathy (BIPN) is one of the most common and severe toxicities of bortezomib resulting in dose reduction or drug discontinuation [2]. The incidence of BIPN varies from 37% for grade 3 to >80% for all grades [2]. Small percentage (~6%) patients can develop painful peripheral neuropathy that usually occurs acutely and takes a long time to resolve [3]. BIPN usually occurs within the first 5 cycles of bortezomib and may resolve between 3 to 48 months after the discontinuation of the drug [2]. Major risk factors for BIPN are cumulative dose of bortezomib and pre-existing peripheral neuropathy from other causes such as diabetes or multiple myeloma. Only 31% (11/35) of patients experiencing grade 3 BIPN had complete symptoms resolution after discontinuing bortezomib [4]. Prospective clinical trial with nerve conduction study suggests that BIPN is a length-dependent, sensory, axonal, large-fiber polyneuropathy [5]. Little is known about the mechanism of BIPN. Animal studies suggest that BIPN may be the result of mild to moderate pathological changes in Schwann cells and myelin, axonal degeneration, and dorsal root ganglia neuron changes [6]. The dorsal root ganglial neuronal cell body changes may result from proteasome inhibitor such as bortezomib, which induces chromatolysis of dorsal root ganglial neurons and causes cytoplasmic accumulation of eosinophilic material [6]. Neurophysiological studies showed that bortezomib induced changes in all three major primary afferent fibers: A*β*, A*δ*, and C [7]. 

The clinical characteristics of BIPN are consistent with dysfunction of sensory nerves causing spontaneous pain, paresthesia (tingling, numbness), hyperalgesia (increase sensitivity to painful stimuli), allodynia (hypersensitivity to non-painful stimuli), and decreased physical activity [8]. Patients may experience neuropathic pain in the stocking and glove distribution with an average pain score being 7.8 out of 10 [7]. They may have distal sensory loss, with the loss in lower extremities being worse than the upper extremities. They may have decreased deep tendon reflexes and changes in proprioception that affects daily activities. In addition, up to 10% of patients with BIPN experience grade 1–3 motor neuropathy [2]. The treatment for BIPN has been limited to symptom management with narcotics, antidepressants, anticonvulsants, and vitamins [2]. Studies suggest that such analgesic regimens usually only produce modest pain relief and are associated with side effects such as sedation, dry mouth, and constipation [2, 3, 7]. Therefore, more effective and less toxic treatments for BIPN are needed. 

The efficacy of acupuncture for treating diabetic peripheral neuropathy and HIV-related peripheral neuropathy has been suggested in a number of clinical trials [9–16]. There have been a number of clinical trials showing the effect of acupuncture in reducing neuropathic pain in cancer patients [17, 18]. One clinical trial showed the effectiveness of auricular acupuncture in treating cancer treatment induced neuropathy [18]. A case series suggested potential efficacy of body acupuncture in treating patients with chemotherapy-induced peripheral neuropathy (CIPN) [19]. To date, there has not been any clinical trial to study the effect of acupuncture in alleviating BIPN symptoms [8]. Here, we report a successful case of using acupuncture to relieve BIPN symptoms.

## 2. Case Report

A 48-year-old African-American man with multiple myeloma presented with multiple bony lesions. Past medical history was significant for hypertension and hyperlipidemia. He was a bus driver. He received induction therapy with bortezomib 1.3 mg/m^2^ IV on days 1, 4, 8, and 11, cyclophosphamide 300 mg/m^2^ IV on days 1 and 8, and dexamethasone 40 mg PO on days of bortezomib. Cycles were repeated every 21 days. After three cycles, he achieved partial remission and underwent autologous peripheral blood stem cell transplantation. He then achieved complete remission. After he finished the third cycle of bortezomib, he developed BIPN with tingling, numbness, and burning pain of feet below the ankle despite being off bortezomib therapy and relatively low cumulative dose of the drug. His symptoms worsened over the next four months to the point that he was not able to stand or walk normally because of the severe pain. He was started on neurontin in escalating doses but was unable to tolerate doses over 1200 mg daily due to side effects, mostly dizziness. He was given morphine sulphate (MS) contin 15 mg PO twice daily with oxycodone 5 mg PO every 4 to 6 hours for breakthrough pain with no significant improvement. Pregabalin 75 mg PO twice daily was added without significant pain relief. His BIPN symptoms persisted with pain, tingling, numbness, and poor quality of life from the pain and pain medications. In addition, he was unable to drive because of pain and side effects of his pain medications. He was referred for acupuncture treatment. 

During the patient's initial visit he had significant paresthesia/in bilateral lower extremities. No autonomic symptoms were reported. His self-reported overall pain score was 8 out of 10 on a 11-point visual analog scale (VAS). Neurological exam showed reduced sensation to light touch, pin, and vibration in distal part of his feet and absent ankle reflexes bilaterally. Muscle strength was five out of 5 throughout with limited lower extremity movement because of severe pain. 

**Figure 1:**
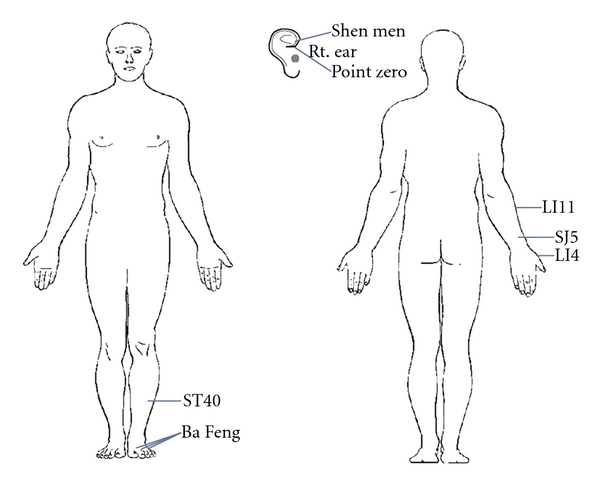
Acupuncture protocol to treat bortezomib-induced peripheral neuropathy.

The patient was treated with an acupuncture protocol, which includes bilateral ear points: shen men, point zero, two additional auricular acupuncture point where electrodermal signal was detected [18], and bilateral body acupuncture points: LI4, SJ5, LI11, ST40, and a set of extraordinary points, Ba Feng (Figure 1). For auricular points, the electrodermal signal was detected through a hand-held auricular acupoint finder (the Pointer-Excell II) where a buzzing sound went off when the electrodermal signal is detected. He was treated once per week for six treatments. He noted that the initial 2 acupuncture sessions gave him pain relief for only a few hours. But the subsequent acupuncture treatments gave him progressively longer pain relief. At the end of his six acupuncture treatments, his self-reported overall pain score went down from 8 out of 10 to 2 out of 10 on VAS. His use of breakthrough pain medication (oxycodone) decreased from 20 to 30 mg daily to 5 to 10 mg daily. He no longer had difficulty walking and standing. He continued acupuncture every other week for four treatments, then once every three weeks for two treatments, and finished with once per month for two treatments. At the end of his 14 acupuncture treatments, he stopped taking MS contin and Oxycodone and was able to go back to work to drive buses. He has been followed every three months and had no recurrent symptoms at last followup, 1 year after the initiation of his symptoms. Throughout his 14 acupuncture treatments, no adverse events related to acupuncture were noted.

## 3. Discussion

This is the first case report on the use of acupuncture in treating multiple myeloma patients with BIPN. Our report suggests that acupuncture is a treatment option for patients experiencing BIPN. Fourteen acupuncture treatments helped the patient to have significant less neuropathic pain, reduced requirement for narcotics, and improved function with minimal side effects. 

The acupuncture protocol was developed from our clinical experience and prior chemo-induced peripheral neuropathy acupuncture research [18, 19]. One randomized, blinded clinical trial showed the effectiveness of auricular acupuncture in treating cancer therapy induced neuropathy [18]. The patients were randomized into one of three arms. One arm received real auricular acupuncture embedded at real ear acupuncture points, which is defined as points where the electrodermal signal is being detected. The other two arms were sham control arms: one received real auricular acupuncture at the placebo points and one received sham acupuncture through auricular seeds at the placebo points [18]. All patients received two courses of real or sham auricular acupuncture in two consecutive months. The needles were left in place until they fell out or were removed at a followup appointment. Their pain intensity measured by VAS at the end of the second month was used to measure the treatment efficacy. This study showed that in the group that received real acupuncture, pain intensity decreased by 36% at the end of 2 months when compared with baseline, whereas it only decreased by 2% in the placebo groups (*P* < .0001) [18]. Moreover, a case series suggested potential efficacy of body acupuncture in treating patients with chemotherapy-induced peripheral neuropathy (CIPN) [19]. Five patients with greater than world health organization grade II CIPN were treated with 6 weekly acupuncture treatments followed by 4 weeks of rest and 6 additional weekly acupuncture treatments. All patients but one finished all 12 acupuncture treatments. All five patients reported improvement in pain, tingling, and numbness. Pain scores decreased from 6–9/10 to 2–3/10 after acupuncture treatments. All patients had reduced analgesic medication intake. Four out of five patients reported persistent symptoms relief at the 6 month followup [19]. 

We observed similar effect in our case report to those aforementioned reports, which is that patient with CIPN responded to acupuncture treatments. Our case suggests that acupuncture may be effective in relieving BIPN symptoms. Although studies have not been able to fully explain the mechanism of acupuncture, it has been proposed that acupuncture works through its effect on neurotransmitters and neurohormones [20–23]. Animal research suggests that acupuncture accomplishes its anesthesia effect by stimulating nerves in the muscle, which then relay the signal to the spinal cord, midbrain, and hypothalamus-pituitary system, which then lead to the release of neurotransmitters and hormones, that is, endorphins and enkephalins [24–26]. Other mechanisms such as activation of descending pain inhibiting pathways, deactivation of the limbic system, cortical cerebral vasodilation causing release of neuropeptide, and inhibition of the release of inflammatory factors have also been suggested to explain the effect of acupuncture analgesia [27–31]. Recent study also showed that electroacupuncture alleviates bone cancer pain by suppressing spinal expression of interleukin-1*β* in glia cells that are involved in the spinal transmission and processing of noxious inputs from the peripheral sites and facilitates pain [32]. It is perceivable that acupuncture reduces BIPN symptoms by suppressing activities of glial cell and stimulating neurohormonal pathways, which increases endorphins release and reduces proinflammatory cytokines. Further study needs to be conducted to explore the role of acupuncture in helping patients suffering from BIPN and its mechanisms of action. 

Our case report is limited in a couple of ways. First, the patient's relief of peripheral neuropathy symptoms may be due to a spontaneous remission rather than the effect of acupuncture. However, based on our clinical experience, once BIPN becomes so severe that it interferes with daily activities, it rarely resolves by itself, and even so, it usually takes a long time (months to years) to do so. Second, the clinical improvement in this patient was primarily documented by subjective end point, the VAS score. Changes in objective end points such as detailed neurological exam and nerve conduction study will help understand how acupuncture worked in reducing BIPN. This case report is the beginning of our endeavor to investigate the role of acupuncture in relieving BIPN. We plan to incorporate more objective measurements such as nerve conduction study into future studies on this topic.
